# The Barriers and Enablers to Implementing Child and Family Health Hubs for Migrant Families

**DOI:** 10.5334/ijic.9847

**Published:** 2026-06-08

**Authors:** Michael Hodgins, Katarina Ostojic, Karen Edwards, Kenny Lawson, Nan Hu, Kim Lyle, Michelle Jubelin, Amit Arora, Kathleen Baird, Ann Dadich, Valsamma Eapen, Amanda Henry, Nick Hopwood, Jane Kohlhoff, Grainne O’Loughlin, Raghu Lingam, Helen Rogers, Shanti Raman, Tania Rimes, Sue Woolfenden, On Behalf of the FDCC Collaborative Group

**Affiliations:** 1 Population Child Health Research Group, School of Clinical Medicine, University of New South Wales, Sydney, Australia; 2 Community Paediatrics Research Group, Faculty of Medicine and Health, University of Sydney, Sydney, Australia; 3 Retired; 4 Health Economics, Psychiatry & Mental Health, University of New South Wales Sydney, Australia; 5 Translational Health Research Institute, Western Sydney University, Sydney, Australia; 6 Child, Youth and Family Primary and Community Health, North Sydney Local Health District, Sydney, Australia; 7 Child, Youth and Family Services, Population and Community Health, South Eastern Sydney Local Health District, Sydney, Australia; 8 School of Medicine, Faculty of Health, Western Sydney University, Campbelltown, Australia; 9 School of Nursing and Midwifery, Faculty of Health, University of Technology Sydney, Sydney, Australia; 10 School of Business, Western Sydney University, Sydney, Australia; 11 Academic Unit of Child psychiatry South West Sydney (AUCS), South Western Sydney Local Health District & Ingham Institute, Sydney, Australia; 12 Discipline of Psychiatry, School of Clinical Medicine, University of New South Wales, Sydney, Australia; 13 The George Institute for Global Health, University of New South Wales, Sydney, Australia; 14 Faculty of Design & Society, University of Technology Sydney, Sydney, Australia; 15 Department of Curriculum Studies, University of Stellenbosch, Stellenbosch, South Africa; 16 Karitane, Sydney, Australia; 17 Discipline of Women’s Health, School of Clinical Medicine, University of New South Wales, Sydney, Australia; 18 Community Paediatrics, South Western Sydney Local Health District, Liverpool, Australia

**Keywords:** hub models, integrated hub, migrant, child, family

## Abstract

Migrant women and children can face significant barriers when accessing child and family health services. Child and family health hubs (hubs) represent a potential solution to simplify pathways between maternity, child, and social care services for migrant populations. However, how these hubs operate specifically for migrants remains underexplored. Our study addresses this gap by exploring what helps and hinders the establishment and delivery of hubs across three different contexts in New South Wales, Australia for migrant populations using a case study approach. While all sites found the hub model appropriate and acceptable, its feasibility varied. Challenges included limited resources, particularly the absence of a dedicated hub coordinator, governance issues, and personnel turnover. Success depended on clear leadership, dedicated coordination, systematic referral pathways, cultural sensitivity, and community engagement. The evidence from this study can be used within other contexts to plan and deliver hubs for migrant and potentially other priority population families with young children.

## Introduction

Migrant women and their children often face barriers when accessing child and family health services [1,2,3,4,5]. Reduced access to child and family health services for migrant mothers can lead to undiagnosed postnatal depression, the early cessation of breastfeeding, and unmet psychosocial support needs, such as those associated with housing instability and domestic violence [6,7,8]. Children from migrant families tend to have their health and development under-monitored compared to others – this can delay the identification of developmental issues and reduce access to early interventions, despite greater needs [9,10,11]. Persistent obstacles to care for these populations include limited English proficiency, insufficient access to basic necessities, cultural adjustment difficulties, fear, stigma, low health literacy, and limited healthcare entitlements or insurance [6,10,12,13,14,15,16,17,18,19,20]. As a result, these populations can experience inequitable access to care, compounded by a fragmented service system with poor communication, unclear referral processes, and service gaps [21].

Child and family health hubs (hereafter hubs) represent a promising approach to address the healthcare needs of priority populations [22,23]. Hubs involve physically co-located or virtually connected health and social services targeted towards child and family health concerns with supported care navigation and shared referral pathways [24,25]. Within these hubs, integration between services is prioritised so that people receive the care they need, when they need it, in ways that are user-friendly, achieve the desired results, and provide value for money [26].

Hubs can simplify pathways between maternity, child and family health, and social care services for mothers and children from migrant backgrounds [27,28,29,30,31]. Evaluating the implementation of complex interventions, such as hubs, is crucial to understand the success or failure of an intervention in a specific context by identifying factors that might moderate its implementation [32,33]. Key factors to operationalise hubs include buy-in and partnership development, establishing hub coordination and navigation, activities, workforce development, funding, and ongoing integration mechanisms [34,35]. However, how hubs are operationalised for migrant populations remains a gap in the literature. Our study addresses this gap by exploring what helps and hinders the establishment and delivery of hubs across three different contexts in New South Wales, Australia for migrant populations.

## Method

### Intervention

Within our study, hubs involved integrating child and family health nursing (CFHN) services and non-government organisations (NGO), including psychosocial support services (e.g., playgroups, domestic violence support, mental health support, early childhood education, family support). In the Australian public health system, CFHN services are typically delivered by state-funded public health services and are administered through geographically defined Local Health Districts (LHDs), which are regional organisations responsible for planning, funding, and delivering hospital and community health services within a defined geographic catchment. The hubs were intended to encapsulate a physical building and a way of facilitating service collaboration, integration, and a community-led approach to local needs. Hubs operated from a host building from which partner community-based or public services were delivered. The implementation of the hub involved the study team fostering integration and collaboration between CFHN and NGO services. The LHD and NGOs contributed staff members to coordinate and implement the hubs, in-kind.

Three study sites were selected based on their infrastructure to support a hub; namely, a building to co-locate services and at least preliminary efforts to integrate health and NGO services. Early work in our study involved co-designing the core components of hubs with the implementing teams during site-specific workshops. These core components included co-located services, established integration processes between health and NGO services, cultural sensitivity for migrant families, and navigation support from maternity services to the hub. Given the limitations of our study timeframe, hubs within this study focused on the implementation of integrated services for migrant and refugee mothers in the first 12 months of their child’s life [31].

### Study design

Our implementation evaluation was embedded within a non-randomised controlled study conducted in the Sydney area in New South Wales (NSW), Australia, between November 2021 and April 2024 (ethics approval number: 2020/ETH03295, Australian New Zealand Clinical Trials registration number: ACTRN12621001088831). Ethics approval for the implementation evaluation was covered by the trial ethics approval. More detail on our study design can be found elsewhere [31,34]. This study was conducted in three LHDs – namely, South Western Sydney, South Eastern Sydney, and Northern Sydney. In these areas, between 38% and 49% of residents spoke a language other than English at home [36].

Using a case study approach, the implementation evaluation assessed the barriers and facilitators when implementing and sustaining a hub model at the three sites. Case study methodology is a research approach that involves an in-depth, contextualised investigation of one or more bounded “cases” such as an individual, group, organisation, event, or program [37,38]. A case study is typically defined as a detailed, systematic examination of a specific case (or small number of cases) over time and within its real-life setting. Case study methodology is well suited to implementation evaluation in health systems research because it can provide insight into the nuances of diverse contexts [37,39]. The method seeks to understand how and why certain processes, decisions, or outcomes occur, emphasising the interplay of context, actors, and mechanisms in an implementation context. Within our study, each of the three participating study sites represented a distinct case to explore the implementation of the hub model. Descriptive qualitative methods were used to capture data within each of the participating case sites.

Our approach was guided by the consolidated framework for implementation research (CFIR), a comprehensive framework designed to ‘offer an overarching typology to promote implementation theory development and verification about what works where and why across multiple contexts’ [40]. Additionally, we aimed to evaluate specific implementation outcomes of acceptability, appropriateness, adoption, coverage, cost, fidelity, and sustainability to the implementation strategy, guided by Proctor and colleagues’ [41] taxonomy.

### Involvement of people with lived experience

The research questions were developed based on qualitative research undertaken with hub participants and community members and service providers in the pilot study. The investigator team has a consumer representative, and consultation was undertaken with local hub partner services. The researchers also consulted multicultural health services, including cultural support workers, to ensure research materials were culturally nuanced. Patients or participants have not directly been involved in the current study design.

### Participants and recruitment

Service providers were purposively recruited (by MH and KE) within the three LHDs at both 6- and 12-months post-trial commencement. The implementation evaluation team extended an email invitation to staff who worked within each hub to participate in an interview or a focus group at a preferred time and location. Mothers and partners were purposefully recruited from the trial participants who had attended the hubs. Questionnaires administered to mothers assigned to the hub during the trial included an item seeking their permission to contact them about an opportunity to participate in an interview, in-person or via telephone. Mothers who provided permission to be contacted were invited to participate in an interview at a preferred time and location. Participants were provided an information sheet and consent form, and the interview process was discussed prior to providing consent and conducting the interview. Service provider participants across the three sites (*n* = 27) included health service providers (10), social service providers (7), project officers supporting implementation (4), health service managers (3), social service managers (2), and a hub coordinator. These participants were spread evenly across Site A (7), Site B (10), and Site C (10). Consumer participants included 14 migrant parents (12 mothers and two fathers) who had attended the hubs in the two sites that proceeded to the trial evaluation. The consumer participants’ countries of birth included Bangladesh, Nepal, Mongolia, Vietnam, and Iraq. Invitations were provided in English and the common language for the area, and all participants were offered interpreter services however the majority declined this and subsequently may have impacted participation rates of families with limited or no English proficiency.

### Data Collection

Participants participated in semi-structured interviews guided by the CFIR [40] and Proctors and colleagues’ [41] implementation outcomes, investigating the barriers and facilitators to implement the hub as part of the trial. Interviews were conducted with service providers and migrant parents who had attended the trial hubs, exploring the experiences of implementing the hub across the sites. All service provider interviews were conducted online via Microsoft Teams™ or Zoom™. Parents participated in an interview (between 10–30 minutes), in-person or via telephone with a research assistant, supported by the first author (MH). Interviews were conducted in English with an interpreter service offered (none of the participants accepted the use of an interpreter).

### Analysis

**Figure 1:**
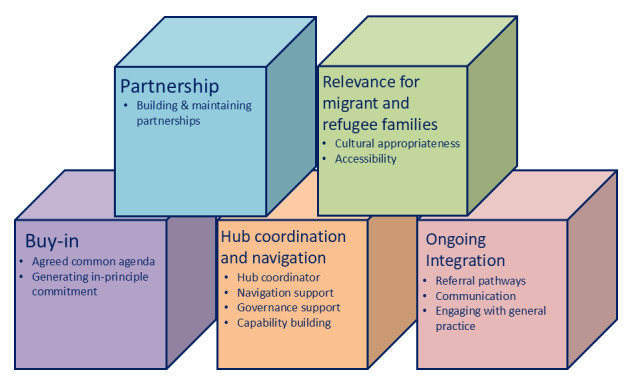
Core Components of a Hub Figure [34].

We managed and analysed the transcripts using NVivo 12©. Interview data were thematically analysed [42]. Our team has identified the core hub components for migrant families and potential strategies to improve implementation success [34]. Core components include the initiation phase activities of buy-in and partnership development, establishing hub coordination and navigation, activities that enhance a hub’s relevance for migrant families, and ongoing integration mechanisms, such as the engagement of same language general practitioners. Analysis involved exploring the core components to implement hubs across the three sites as separate case studies (see Figure 1).

The analysis was also guided by Proctors and colleagues’ [41] implementation outcomes, including acceptability, appropriateness, feasibility, and adoption. Two researchers conducted the analysis (MH, KE), which involved familiarisation with the dataset, generating initial codes via line-by-line coding, and collating data relevant to each code. An initial coding framework that reflected the core components to implement hubs as well as implementation outcomes was developed initially by two authors (MH, KE) and refined through discussion with other authors (KO, TR, SW). Excerpts of interview data are presented throughout the results section as key quotes illustrating the findings from our analysis.

To supplement interview data, a bespoke hub intervention log was developed to determine the fidelity of adoption of the hub components within each site, to be completed by site project officers at both the midpoint and end of the trial (Tables 1 and 2). The intervention log was guided by our logic model and represented the components of the hub model to be delivered within each site [34]. The intervention log collected data on the implementation of the intervention components within two of the three sites that progressed to the trial phase of the research (Sites B and C). Fidelity tool data are presented within each case study.

**Table 1:** Site B Fidelity Log.


HUB PARTNERED HEALTH AND NGO SERVICES	INTEGRATION OF SERVICES	CULTURAL SENSITIVITY	NAVIGATION MODEL

Child and family health nurse Nutrition Clinic Community Paediatrics NGO Child health early intervention and prevention service NGO Parent support service NGO Music therapy Speech Pathology NGO Child psychological therapies NGO Family mental health service	**Referral Pathways between services within hub**	**Training**	None specified

	Referral pathways documented (supplementary file)	None identified	

	**Communication pathways between services within hub**	**Indicate use of interpreters**	

	Monthly site meeting set up as part of the trial No joint case review meeting No joint client intake meeting	Free access to interpreters	

		**Appropriate resources**	

		No in-language resource identified	

	**Shared Training between services within hub**	**Consumer Engagement/codesign with local communities**	

	Common agenda training	None identified	

	**Shared Resource between services within hub**		

	Shared building Shared security		

	**Shared Strategy between services within hub**		

	No documented joint strategy		


**Table 2:** Site C Fidelity Log.


HUB PARTNERED HEALTH AND NGO SERVICES	INTEGRATION OF SERVICES	CULTURAL SENSITIVITY	NAVIGATION MODEL

**Child and family health nurse (Health)** **Childcare Centre** **Community Paeditrican** **Public School** **School Nurse** **Two supported Play groups** **NGO parenting and family service** **NGO disability service for children and young people** **NGO Navigator** **Speech therapist** **Occupational therapist**	**Referral Pathways between services within hub**	**Training**	Partner NGO provide service navigation support for the hub as part of their funding

	Referral pathways documented (Bespoke processes to support integration e.g. “warm” referrals and refer feedback)	Trauma informed care Working in Culturally Diverse Contexts Course (Health staff only) Culturally responsive practice workshop	

	**Communication pathways between services within hub**		

	Governance structure: working group and leadership group made up of health and NGO partners Referral feedback provided No joint case review meeting No joint client intake meeting	**Indicate use of interpreters**	

		Free access to interpreters	

		**Appropriate resources**	

		Multi-language welcome signs Hub brochure in 5 community languages Links to translated resources in confirmation emails; explained at first appointment with interpreter if required.	

	**Shared Training between services within hub**		

	Developmental surveillance Trauma informed care Common agenda		

	**Shared Resource between services within hub**	**Consumer Engagement/codesign with local communities**	

	Shared building Shared digital resources	QI survey of Child, Youth and Family Services	

	**Shared Strategy between services within hub**		

	Strategic Planning Workshops Shared vision documented in hub brochure and referral guidelines		


## Results

The results are organised by site, exploring how implementation of hub core components proceeded. In each section, we summarise how each site achieved the core components with select quotes and an overall summary of the hub site’s acceptability, appropriateness, feasibility, and adoption. Additional quotes are provided in supplementary file 1. Given the links between how buy-in and partnership developed within each site, these core components were integrated within our analysis.

### Site A: A Nascent Hub

#### Description of the Hub

Site A was based in an area where the highest proportion of residents born in a country other than Australia were born in China, Korea, India, Philippines, and Hong Kong. In the 2021, national data indicated that 59% of families reported using a language other than English at home, with the top languages being Mandarin, Cantonese, Korean, Arabic, and Hindi [36]. The hub location was a three-story building, purpose-built and managed by the local council. The expressed intention for the building was to co-locate child and family services to collaborate on projects and referral pathways, however this initiative was still in its infancy with limited progress towards a fully functioning hub model. The use of the name, hub, for the building was confusing during interviews as people tried to differentiate between the hub building, managed by the council, and the hub as a collaborative integrated cross-agency model of practice. The council’s role in owning and managing the building influenced participants’ sense of control when they discussed developing an integrated hub in the building. The services based in or using the building included a CFHN service and child and family health centre, managed by the LHD, and NGO services that offered child and family related services, domestic and family violence support, community housing, early childhood education, and relationship counselling.

#### Buy-In and Partnership

Participants had a shared understanding of the challenges faced by the community. While they supported the concept of a hub, achieving agreement between stakeholders on what a hub is and what it should do within the site had yet to be achieved. Some of the groundwork was in place for establishing trusted relationships to move from co-located or referred services to an integrated hub:

*We’re evolving in the community… to a service that’s much more focused on vulnerable families. So, I think it fits in the vulnerable family space… these families really, really need our help (Site A health provider)*.

*But I could imagine the childcare centre, CFH, and us in this kind of governance group whatever you want to call it. Because at the end of the day, we’re all wanting the same, we’re wanting better outcomes (Site A NGO manager)*.

Informal partnerships existed between the CFHN service and the main NGO based in the facility. While staff members expressed a willingness to develop stronger partnerships, they had not agreed on or established formal partnerships. For example, there were no formal cross-partner governance structures at senior management level established to support resourcing, change management, and implementation of the hub:

*Even though we’ve got this amazing service, [an NGO]… on the top floor… [another NGO] on the next floor, we’ve also got an amazing supportive playgroup on the ground floor. We’ve also got housing here as well. Child and family health is a little bit behind a closed door and very clinician dependent on how and the relationship that they have with those other services within the same building (Site A health service manager)*.

#### Hub Coordination and Navigation

Participants at Site A acknowledged the importance of hub coordination and navigation and agreed that the next priorities to establish a hub were a dedicated hub coordinator and navigator:

*You need a coordinator. And part of that role of that coordinator would be working with those organisations to identify what the emerging need is, ensure that everyone’s contracts are up to date etc. (Site A NGO manager)*.

While there was no identified navigator role, the concept of a concierge role to support client wayfinding was discussed by the main NGO.

#### Relevance for Families

There were promising elements within Site A to support a service for migrant families, including in-language resources and culturally-relevant parenting groups. Participants highlighted the need for future work to develop a friendly and welcoming environment for migrant families, improve links with general practitioners (GPs), and work towards dedicated consumer engagement processes:

*I think that for a lot of [migrant] families… there’s a trusting relationship with a GP; we know from our GP pilot that some culturally linguistically diverse families will travel suburbs and suburbs away to see a general practitioner who speaks their language (Site A health service manager)*.

*Can we make [the space] more appropriate, more approachable, and warmer, and maybe some flags out the front – a bit of life and energy, have a concierge at the front desk to help you navigate your way through the building? (Site A NGO manager)*.

Participants also expressed support for the concept of a single entry into the hub, via physical and virtual models.

#### Ongoing Integration

Agency protocols to share client information between the nascent hub partners presented perceived and actual barriers to collaborate with other agencies in an integrated model of care. While ad hoc ‘warm referrals’ between CFHN and the main NGO occurred, pathways that support warm referrals and collaborative care had yet to be agreed, systematised, and supported by an integrated care model:

*We’ve been co located in that building for 9 or 10 years. And I don’t see the type of integration at a client level, we would hope to achieve. I think there’s a lot of good intent, but I don’t think our service is particularly integrated at the client level (Site A health service manager)*.

Some participants also noted the challenges around different ways of working for staff members starting within a hub model, which required dedicated support to overcome.

In summary, although a hub was not adopted as intended at Site A, participants agreed that a hub for migrant families was appropriate and acceptable. Most participants were willing to discuss the development of a hub. Existing relationships can be built on, especially between the NGO and LHD. Participants viewed the barriers as surmountable with dedicated resourcing.

### Site B: Established

#### Description of Hub

Site B was based in an area where the highest proportion of residents born in a country other than Australia were born in Vietnam, Lebanon, Iraq, New Zealand, and Fiji.^1^ In the 2021, 53.8% of families reported using a language other than English at home, with the top languages being Arabic, Vietnamese, Samoan, Khmer, and Hindi [36]. Our study provided an opportunity for established NGO and LHD care providers to develop a hub for families with children, aged 0 to 5 years, within an NGO-owned and -managed building. The NGO that owned the building accommodated child and family-oriented social services, including services for families with older children, and the LHD CFHN service. The site incorporating both NGO and LHD services has been in operation for 25 years. At the time of the project, other services co-located in the building supported a range of individuals and families across multiple social care services. Services relevant to the 0 to 5 age group included CFHN, dietetics, paediatrics, family support, speech therapy, parenting support, and music therapy (see Table 1).

#### Buy-In and Partnership

Participants indicated there was still work to be done in developing an agreed and shared approach to address health and wellbeing needs, given the lack of formalised commitments between partners. Participants reported that the COVID-19 pandemic and staff member changes impacted relationships between service providers. Site B faced specific challenges during the implementation of the hub, with hub leaders from the LHD and NGO leaving their roles. One challenge was the limited relevance of co-located NGO services for infants, which was the focus of these hubs during the trial phase of the research. Most existing NGO services were targeted at older children:

*Not all of our partners were appropriate to be part of the project, because one of them only delivers to primary school aged and the upper primary age children. So, they weren’t even sort of considered as part of it… So, I don’t think things were overly clear to all of those programs as to why that we’re being asked to be part of [a hub] (Site B NGO manager)*.

Also, service providers and family members noted that families typically preferred GPs who spoke their birth country language, instead of the CFHN service, limiting the appropriateness of the hub for this area. The locus of control of the hub fell to the NGO, with some health providers indicating they felt like ‘*contractor[s]*’, not fully integrated into hub operation. While NGO participants indicated positive steps in developing relationships with health service staff, they also indicated more work was needed to build relationships and shared understanding:

*It’s [the NGOs] building*, *and they do their thing. And I don’t think they really want to listen to what we have to say – in a nice way. They just feel that they’re not required for the child and family health service. They think the ages that they see, the kids, have nothing to do with child and family health (Site B health service provider)*.

The Site B hub partners each provided services to address their client group’s health and wellbeing needs. These client groups did not necessarily align across the different services. Given the challenges achieving initial buy-in and shared understanding between partners, according to participants, this affected partnerships within Site B.

#### Hub Coordination and Navigation

Site B lacked a dedicated hub coordinator and referral pathways. As part of implementation, the Site B project officer assumed the coordinator role, arranging regular meetings to aid case review, relationships between health and NGO providers, and a shared referral pathway. Initially, the referral pathway and regular meetings began building an integrated approach, with health service and NGO staff indicating this was a positive development for the hub. However, the project officer vacated the role partway through implementation, causing the coordination of hub activities to ‘fall apart’.

*I think [the project officer]…really had tried to hold that role together. And as I said, it really seemed to fall apart once she had moved on from the project because she had coordinated everybody*, *trying to coordinate those meetings, and she’d done an amazing job at it. That would certainly be something that would be needed ongoing to make a successful hub is to have a coordinator to make that happen (Site B NGO manager)*.

#### Relevance for Families

Dedicated work to improve the service for migrant families was underway within site B. However, all sites lacked a systematic approach to hear from community members and identify their priorities. This might partly explain why some service providers and family members noted that service relevance for migrant families was hampered:

*I think that the emerging populations move into [Site B] because of the cheapness of rent, [which] then leads itself to the inequities of trying to access interpreters at those times that they’re being seen… And also, the availability of a GP that might speak their own language that might be open five days a week… particularly with the culturally and linguistically diverse populations (Site B health service provider)*.

*We went to the GP check-up for six months and vaccination. And we’ve been sick lately. So, we just call GP and check up on him. [I chose to see the GP for the 6 months check] because I don’t have any appointment [with the hub]… When you go to GP, you have the vaccination and do all the checks all over again. I think that like a best for me to go to [the GP]. (Site B mother)*.

Participants indicated that waiting lists for specialist services, such as paediatricians, encouraged families to access private, rather than public services. As with Site A and Site C, while GPs were identified as necessary partners in the hub, a process to effectively engage them had not been developed and tested.

#### Ongoing Integration

Long established undocumented referral pathways existed between some service providers in the hub. As part of hub development, formalised referral pathways had been developed for the Site B hub. Services were delivered by individual providers, as usual, with future priorities that included working in a collaborative model:

*No, I don’t have much to do with them at all. It’s been a little bit hard for me to attend all their meetings. I did go to one recently, and I haven’t really had a lot of referrals from them (Site B NGO provider)*.

In summary, while there was buy-in for the hub and some hub coordination and navigation capacity, the feasibility of delivering a hub was hindered by staff member changes and limited resources. Families found the hub feasible to attend, but some indicated a preference for attending a GP. Key priorities for Site B included the co-location of early childhood services, a dedicated hub coordinator, further development of referral pathways, and regular hub governance meetings.

### Site C: An established Hub

#### Description of Hub

Site C was located in an area with a high migrant population. The highest proportion of residents was born in a country other than Australia, including Nepal, China, North Macedonia, Bangladesh, and Philippines.^2^ In 2021, 72.8% of families reported using a language other than English at home, with the top languages being Nepali, Mandarin, Macedonian, Arabic, and Cantonese [36]. Site C was a combined physical and virtual hub in partnership with NGOs and government agencies that operated from their own facilities. A facility on the grounds of the local public school (also a hub partner) was used by CFHN and visiting services. This hub had evolved since 2015, when a pilot project was established, locating the CFHN in the NGO-supported playgroups to increase access to child developmental checks for migrant families. The hub included partners from multiple NGOs, including early childhood education and family support services, a local school, allied health services, the Department of Education, and the Department of Communities and Justice (see Table 2).

#### Buy-In and Partnership

Service providers expressed a collective or mutual understanding of the additional disadvantage experiencedin this area with a high migrant population. They recognised the value of a hub for addressing this:

*There’s a high need, higher need in this area for support for children, preschoolers, especially that might have delays in different parts of their development that they need extra support for before they start school (Site C health service provider)*.

Having commenced with an in-principle commitment to the hub, participants within Site C noted that the hub had progressed formalised agreements, as also noted in their fidelity log (Table 2).

The hub built on existing relationships, which had expanded as other services providers recognised the advantages of an integrated model of care. Participants noted that partnerships with partners required regular contact, understanding their role in the hub, and having clearly identified common goals and priorities. The number of partners in the hub had increased over time, with the inclusion of more early childhood education and disability support services. It was important to ensure the vision and purpose were common to all partners. Given the growing number of partners, a systematised approach to clear and inclusive communication was required, from senior managers to client-facing service providers:

*We now have started to introduce a… it’s not an MOU [memorandum of understanding], it’s like a service agreement… it basically really just outlines what, if you come on as a partner, this is what it actually means (Site C hub coordinator)*.

A leadership group was established to govern the hub with a representative from all partner organisations expected to attend meetings. The leadership group identified and acted on inter-partner issues. Although there were essential agency protocols that did not align among partners, at a local level hub partners and clinicians reported adjusting to these in their work with clients.

#### Hub Coordination and Navigation

The coordinator’s role was described as essential to develop and maintain the hub. The role included making decisions about the functioning of the hub, managing partner relationships, dealing with hub logistics, and managing issues. The Site C hub had multiple entry points via individual agencies and a dedicated entry point via a local NGO for families not already engaged with one or more of the hub partners. Continuity of care through a dedicated CFHN who acted as a navigator was a component of the model. Participants reported these model components – the coordinator, the entry points, and the navigator – were instrumental in the continued engagement of mothers who accessed child healthcare:

*You need someone who is in tune with all the subtleties that often are completely missed. I think without that everyone is busy, and everyone gets back into their own organisations. I think without someone who brings you together all the time it’s hard to keep the momentum going (Site C NGO manager)*.

#### Relevance for Families

Hub partners reported actively seeking engagement with local communities through a variety of approaches. Participants noted: the hub was a key point for further connection with health and social care services, especially for migrant families; and the importance of having a culturally-connected navigator for this population. Clinicians reported engaging flexibly with families for appointment times and the use of technology in recognition of their life stresses as well as language, and cultural preferences:

*Making sure that women and families have a choice of what they want to access and when and that’s part of a mapping exercise. And the navigator once again supports them to say what services are provided at the different locations and their benefits and sorting the referral (Site C health service manager)*.

There were service gaps the hub strived to address, including access to culturally relevant GPs, social work services, and improving waiting times for allied health specialists.

#### Ongoing Integration

According to some participants, warm referrals distinguished the hub from more traditional service delivery models. Warm referrals required time and administration, which should be factored into resourcing. This included built-in feedback loops within referral pathways, i.e. engaging in a two-way conversation between services to ‘*send*’ and ‘*catch*’ hub families:

*I knew that there was collaboration and referrals from partners in the hub, but I didn’t realise how much administration work was involved and feedback, which is a very positive thing (Site C health service provider)*.

*Part of that commitment is it’s about warm referrals. It’s about giving feedback. It’s about having a key person within each of those partner organisations that you can reach out to at any time and ask a question. I think that’s really important (Site C hub coordinator)*.

While participants recognised the importance of GP engagement with families to support integrated care, they noted there was still work to be done in engaging with general practices in a partnership that works within their business models and supports outcomes for families. Participant described formal and informal multidisciplinary conferences between partners and sharing client information (with consent) as business-as-usual. Some reported that they were comfortable to ask hub partners for advice to harness their expertise. Information systems across partners remained unconnected, which hindered the sharing of client information, a persistent problem given the nature of siloed information systems in Australia.

In summary, the Site C hub was viewed as acceptable and appropriate across the service providers, managers, and families who were interviewed. While partners had changed within Site C, participants noted that the hub remained viable, reflecting the appropriateness of established processes. Participants within Site C reported ongoing challenges surrounding consumer and community engagement and partnership with general practice. Families found the hub feasible to attend.

#### Findings Across Sites

Community members and service providers deemed a hub model as appropriate and acceptable; however, the feasibility of hub delivery varied. Hub feasibility was impacted by limited resources, particularly the absence of a dedicated hub coordinator, governance structures, structures to buffer the loss of key personnel, as seen within Site B. Feasibility was enhanced in Site C with the hub coordinator, formalised governance, and clear referral pathways contributing to its success.

In all three sites, the hub coordinator and navigator roles were crucial for hub functionality. In Site A, there was no allocated coordinator role, and the Site B project officer’s departure impacted hub coordination. While Site C had a hub coordinator, the increased number of partners and continued issues around information sharing made it evident that the role was vital for hub success. In Sites A and B, referral pathways were still in development, with limited integration of referral pathways among partners. This highlights the importance of standardised, integrated referral mechanisms for successful hub implementation. Site C had made progress in this area, with warm referrals and multidisciplinary conferences becoming routine, though challenges with system integration and information sharing persisted. All sites recognised the importance of culturally relevant services, such as in-language resources and the need for community engagement with ongoing efforts were required to address gaps in services.

## Discussion

Our research explored the implementation of hubs for migrant families across three sites in Sydney, Australia, providing a comparative, real-world examination of how integrated hub models operate at different stages of maturity within the same policy and service context. Hubs were viewed as appropriate and acceptable across all three sites; however, the success of their implementation varied demonstrating that acceptability alone is insufficient for effective integration. This study advances existing literature by empirically mapping variation in hub implementation to established levels of service integration, rather than treating hubs as a uniform intervention.

The sites in our study represent varying levels of integration, reflecting the intensity levels as outlined by Heath and colleagues’ [43] intensity levels of service integration. Heath and colleagues identified six intensity levels of integrated care. The first two levels are grouped together as ‘Coordinated Care’ and involve basic communication and distant collaboration. The next two are grouped together as ‘Co-located Care’ and focus on onsite collaboration, with level four including some system integration. The final two are grouped together as ‘Integrated Care’ and involve close collaboration and full practice transformation. Site A reflected a co-located rather than integrated care; Site B reflected steps towards integration; and Site C reflected a comprehensively integrated service. Sites A and B faced ongoing challenges related to coordination, integration, and service relevance, with participants expressing the need for formal partnerships, governance structures, and resourcing to overcome these barriers. While Site C had a more mature and integrated model, challenges persisted around certain service gaps and stakeholder engagement. Importantly, our findings highlight that hub sustainability requires ongoing relational work, with continued effort needed to maintain partnerships and periodically revisit shared goals beyond initial buy-in and establishment phases.

This study extends prior hub literature by demonstrating how previously identified implementation components—such as governance, coordination, and time for practice change—interact dynamically across different levels of integration. Consistent with existing evidence, child and family hub literature emphasises the importance of a dedicated hub coordinator, engaged governance, and organisation-wide commitment [35]. Other work has shown the importance of navigation support, workforce development, clear referral pathways, and partnerships for hub development [44]. Our previous work has explored the need for effective hub governance and partnerships, overseen by a hub coordinator with authority and decision-making power for successful implementation [34].

### Lessons learned

Our findings underscore the importance of leadership and coordination in multiagency collaboration. Effective collaboration in integrated service delivery models depends on a central coordinator who oversees operations, builds relationships, and ensures that partners remain focused on the shared goals of the hub [45]. These coordinators require support, including dedicated training across partnering organisations and relevant policies and procedures compiled into one reference resource [46]. Additionally, GP engagement remains a clear priority for ongoing hub relevance for migrant families. Trust between health service providers and culturally and linguistically diverse families is critical, with families more likely to engage with services when they feel heard, respected, and cared for [47,48]. Many families in this study preferred GPs with whom they had established trust and shared language and cultural backgrounds. A recent example of integrated child healthcare between general practice and secondary paediatric care from the United Kingdom, showed that some secondary outcomes, including care quality, improved [49]. However, given that most child health outcomes showed no difference, more time might be required to observe sustained improvement. Future work exploring hubs for migrant families cannot ignore GPs as a foundational element of their care pathway.

Overall, this study advances understanding of how and why hub implementation succeeds or falters across levels of integration. The findings offer practical guidance for policymakers and service planners by identifying the organisational conditions, partnership structures, and primary care linkages needed to move beyond colocation towards sustainable integrated hubs. Dedicating attention to formal partnerships, governance structures, and resourcing to overcome implementation barriers is crucial to ensure implementation success. Additionally, to ensure the successful adoption of hubs for migrant families, future planning must incorporate strong links with existing primary care networks that are relevant to families.

### Limitations

Several limitations should be considered when interpreting these findings. Although the study used defined sampling and recruitment strategies, developed nonEnglish materials, and offered interpreter support, all data collection was conducted in English and no participants used interpreters. Consequently, newly arrived migrant and refugee families with limited English proficiency were underrepresented, despite often facing the greatest barriers to service access and engagement. Familyreported implementation outcomes—particularly access, acceptability, and perceived coverage—therefore reflect the perspectives of Englishproficient families rather than the full diversity of migrant and refugee populations. Future research should prioritise multilingual recruitment, explore barriers to interpreter use, and differentiate refugee and nonrefugee migrant experiences. Additionally, parent participants were typically caring for very young children, often alongside other caregiving responsibilities, which limited interview time and may have constrained data depth despite efforts to accommodate participation.

## Conclusion

Hubs are emerging as a valuable support mechanism for migrant families. However, more work is needed to establish effective ways to implement and sustain hub models for this population. Our findings show that the success of hub models depends on clear leadership, dedicated coordination roles, and systematic approaches to referral pathways, cultural sensitivity, and community engagement.

## Additional File

The additional file for this article can be found as follows:

10.5334/ijic.9847.s1 Additional quotes. Site A data to Site C data.

## Footnotes

https://www.abs.gov.au/census/find-census-data/quickstats/2021/SAL12628.

https://www.abs.gov.au/census/find-census-data/quickstats/2021/SAL13394.

## References

[1] World Health Organization. Common health needs of refugees and migrants: literature review. Geneva: IGO LCB-N-S; 2021.

[2] Rogers HJ, Ao CSH, Henry A. Perspectives of women and partners from migrant and refugee backgrounds accessing the Cross Cultural Worker Service in maternity and early childhood services—a survey study. BMC Health Services Research. 2023;23(1):1233. DOI: 10.1186/s12913-023-10194-3 37946230 PMC10636916

[3] Rogers HJ, Hogan L, Coates D, Homer CSE, Henry A. Cross Cultural Workers for women and families from migrant and refugee backgrounds: a mixed-methods study of service providers perceptions. BMC Women’s Health. 2021;21(1):222. DOI: 10.1186/s12905-021-01368-4 34044833 PMC8161620

[4] Iqbal M, Walpola R, Harris-Roxas B, Li J, Mears S, Hall J, et al. Improving primary health care quality for refugees and asylum seekers: A systematic review of interventional approaches. Health Expect. 2022;25(5):2065–2094. DOI: 10.1111/hex.13365 34651378 PMC9615090

[5] Pangas J, Ogunsiji O, Elmir R, Raman S, Liamputtong P, Burns E, et al. Refugee women’s experiences negotiating motherhood and maternity care in a new country: A meta-ethnographic review. International Journal of Nursing Studies. 2019;90:31–45. DOI: 10.1016/j.ijnurstu.2018.10.005 30583266

[6] Fellmeth G, Fazel M, Plugge E. Migration and perinatal mental health in women from low- and middle-income countries: a systematic review and meta-analysis. BJOG. 2017;124(5):742–752. DOI: 10.1111/1471-0528.14184 27320110

[7] Council of Australian Governments Health Council. The Australian National Breastfeeding Strategy: 2019 and Beyond; 2019.

[8] Goldfeld S, D’Abaco E, Bryson H, Mensah F, Price AM. Surveying social adversity in pregnancy: The antenatal risk burden experienced by Australian women. Journal of paediatrics and child health. 2018;54(7):754–760. DOI: 10.1111/jpc.13860 29442394

[9] Gallegos A, Dudovitz R, Biely C, Chung PJ, Coker TR, Barnert E, et al. Racial Disparities in Developmental Delay Diagnosis and Services Received in Early Childhood. Acad Pediatr. 2021;21(7):1230–1238. DOI: 10.1016/j.acap.2021.05.008 34020100 PMC9169674

[10] Woolfenden S, Eapen V, Jalaludin B, Hayen A, Kemp L, Dissanyake C, et al. Prevalence and factors associated with parental concerns about development detected by the Parents’ Evaluation of Developmental Status (PEDS) at 6-month, 12-month and 18-month well-child checks in a birth cohort. BMJ open. 2016;6(9):e012144. DOI: 10.1136/bmjopen-2016-012144 PMC5020845 27609853

[11] Hirai AH, Kogan MD, Kandasamy V, Reuland C, Bethell C. Prevalence and variation of developmental screening and surveillance in early childhood. JAMA pediatrics. 2018;172(9):857–866. DOI: 10.1001/jamapediatrics.2018.1524 29987317 PMC6143066

[12] Almeida LM, Caldas J, Ayres-de-Campos D, Salcedo-Barrientos D, Dias S. Maternal healthcare in migrants: a systematic review. Maternal & Child Health Journal. 2013;17(8). DOI: 10.1007/s10995-012-1149-x 23334357

[13] World Health Organization. Promoting the health of refugees and migrants: Framework of priorities and guiding principles to promote the health of refugees and migrants. Geneva: WHO Secretariat; 2017. p. 1–4.

[14] Higginbottom G, Morgan M, Alexandre M, Chiu Y, Forgeron J, Kocay D, et al. Immigrant women’s experiences of maternity-care services in Canada: a systematic review using a narrative synthesis. Syst Rev. 2015;4(13):13. DOI: 10.1186/2046-4053-4-13 PMC4506414 26187687

[15] Correa-Velez I, Ryan J. Developing a best practice model of refugee maternity care. Women Birth. 2012;25(1):13–22. DOI: 10.1016/j.wombi.2011.01.002 21315675

[16] Henderson S, Kendall E. Culturally and linguistically diverse peoples’ knowledge of accessibility and utilisation of health services: exploring the need for improvement in health service delivery. Aust J Prim Health. 2011;17(2):195–201. DOI: 10.1071/PY10065 21645477

[17] Heslehurst N, Brown H, Pemu A, Coleman H, Rankin J. Perinatal health outcomes and care among asylum seekers and refugees: a systematic review of systematic reviews. BMC Medicine. 2018;16(1). DOI: 10.1186/s12916-018-1064-0 PMC5996508 29890984

[18] Boyle JA, Willey S, Abbasova G. Supporting better outcomes for migrant and refugee women. O&G Magazine [Internet]. 2018;20(1). Available from: https://www.ogmagazine.org.au/20/1-20/better-outcomes-migrant-refugee-women/.

[19] Small R, Roth C, Raval M, Shafiei T, Korfker D, Heaman M, et al. Immigrant and non-immigrant women’s experiences of maternity care: a systematic and comparative review of studies in five countries. BMC Pregnancy Childbirth. 2014;14(152):1–17. DOI: 10.1186/1471-2393-14-152 24773762 PMC4108006

[20] Woolfenden S, Posada N, Krchnakova R, Crawford J, Gilbert J, Jursik B, et al. Equitable access to developmental surveillance and early intervention – understanding the barriers for children from culturally and linguistically diverse (CALD) backgrounds. Health Expectations. 2015;18(6):3286–3301. DOI: 10.1111/hex.12318 25510182 PMC5810693

[21] Council of Community Pediatrics., Chilton LA, Handal GA, Paz-Soldan GJ, Granado-Villar DC, Gitterman BA, et al. Providing Care for Immigrant, Migrant, and Border Children. Pediatrics. 2013;131(6):e2028–e2034. DOI: 10.1542/peds.2013-1099 23650300

[22] Council on Community Pediatrics. Committee on Native American Child Health. Health Equity and Children’s Rights. Pediatrics. 2010;125(4):838–849. DOI: 10.1542/peds.2010-0235 20351009

[23] The Royal Australasian College of Physicians. Inequities in Child Health Position Statement. Sydney, Australia; 2018 May.

[24] National Academies of Sciences E, Medicine. Integrating Social Care Into the Delivery of Health Care: Moving Upstream to Improve the Nation’s Health. Washington, DC: National Academies Press; 2019. 31940159

[25] Moore T. Using place-based approaches to strengthen child wellbeing. Developing Practice: The Child, Youth and Family Work Journal. 2014;40:40.

[26] World Health Organization. Integrated health services—what and why? Technical Brief No.1. World Health Organization; 2008.

[27] Glover J, Samir N, Kaplun C, Rimes T, Edwards K, Schmied V, et al. The effectiveness of place-based interventions in improving development, health and wellbeing outcomes in children aged 0–6 years living in disadvantaged neighbourhoods in high-income countries – A systematic review. Wellbeing, Space and Society. 2021;2. DOI: 10.1016/j.wss.2021.100064

[28] Edwards B, Mullan K, Katz I, Higgins D. The Stronger Families in Australia (SFIA) Study: Phase 2; 2014.

[29] Edwards K, Rimes T, Smith R, Fernandez R, Stephenson L, Son J, et al. Improving Access to Early Childhood Developmental Surveillance for Children from Culturally and Linguistically Diverse (CALD) Background. Int J Integr Care. 2020 Apr;20(2). DOI: 10.5334/ijic.4696 PMC7181950 32346361

[30] Edwards K, Fernandez R, Rimes T, Stephenson L, Smith R, Son J, et al. Happy, healthy, ready – working with early childhood non-government organisations for developmental surveillance for vulnerable children. Australian Journal of Advanced Nursing. 2020;37(4):37–46. DOI: 10.37464/2020.374.277

[31] Hodgins M, Ostojic K, Hu N, Lawson KD, Samir N, Webster A, et al. Study protocol for a real-world evaluation of an integrated child and family health hub for migrant and refugee women. BMJ open. 2022;12(8):e061002. DOI: 10.1136/bmjopen-2022-061002 PMC10439340 36041760

[32] May C, Finch T, Mair F, Ballini L, Dowrick C, Eccles M, et al. Understanding the implementation of complex interventions in health care: the normalization process model. BMC Health Services Research. 2007;7(1):148. DOI: 10.1186/1472-6963-7-148 17880693 PMC2089069

[33] Skivington K, Matthews L, Simpson SA, Craig P, Baird J, Blazeby JM, et al. A new framework for developing and evaluating complex interventions: update of Medical Research Council guidance. bmj. 2021;374. DOI: 10.1136/bmj.n2061 PMC8482308 34593508

[34] Hodgins M, Ostojic K, Rimes T, Edwards K, Lawson K, Fonseka M, et al. The Building Blocks for Successful Hub Implementation for Migrant and Refugee Families and Their Children in the First 2000 Days of Life. Health Expectations. 2025;28(1):e70082. DOI: 10.1111/hex.70082 39792575 PMC11721473

[35] Calik A, Liu HM, Montgomery A, Honisett S, Van Munster K-A, Morris T, et al. Moving from idea to reality: The barriers and enablers to implementing Child and Family Hubs policy into practice in NSW, Australia. Health Research Policy and Systems. 2024;22(1):83. DOI: 10.1186/s12961-024-01164-0 39010121 PMC11247851

[36] Australian Bureau of Statistics. Cultural diversity: Information on country of birth, year of arrival, ancestry, language and religion; 2021.

[37] Stake R. Case study research. Cham: Springer; 1995.

[38] Yin RK. Case study research and applications. Thousand Oaks, CA: Sage; 2018.

[39] Sibbald SL, Paciocco S, Fournie M, Van Asseldonk R, Scurr T. Continuing to enhance the quality of case study methodology in health services research. Healthcare management forum. 2021;34(5):291–296. DOI: 10.1177/08404704211028857 34227408 PMC8392758

[40] Damschroder LJ, Aron DC, Keith RE, Kirsh SR, Alexander JA, Lowery JC. Fostering implementation of health services research findings into practice: a consolidated framework for advancing implementation science. Implement Science. 2009;4(1):50. DOI: 10.1177/08404704211028857 PMC2736161 19664226

[41] Proctor E, Silmere H, Raghavan R, Hovmand P, Aarons G, Bunger A, et al. Outcomes for implementation research: conceptual distinctions, measurement challenges, and research agenda. Administration and Policy in Mental Health and Mental Health Services Research. 2011;38(2):65–76. DOI: 10.1007/s10488-010-0319-7 20957426 PMC3068522

[42] Braun V, Clarke V. Using thematic analysis in psychology. Qual Res Psychol. 2006;3(2):77–101. DOI: 10.1191/1478088706qp063oa

[43] Heath BWRP, Wise Romero P, Reynolds K. A Standard Framework for Levels of Integrated Healthcare. Washington, D.C.: SAMHSA-HRSA Center for Integrated Health Solutions; 2013.

[44] Honisett S, Cahill R, Callard N. Child and family hubs: an important ‘front door’ for equitable support for families across Australia: National Child and Family Hubs Network; 2023.

[45] Lasker RD, Weiss ES. Broadening participation in community problem solving: A multidisciplinary model to support collaborative practice and research. Journal of Urban Health. 2003;80(1):14–47. DOI: 10.1093/jurban/jtg014 12612096 PMC3456118

[46] Elliott IC, Sinclair C, Hesselgreaves H. Leadership of integrated health and social care services. Scottish Affairs. 2020;29(2):198–222. DOI: 10.3366/scot.2020.0316

[47] Roberts N, Jacmon H, Scanlon B, Battersby C, Buttrum P, James C. How can we meet the needs of patients, their families and their communities? A qualitative study including clinicians, consumer representatives, patients, and community members. BMC Health Serv Res. 2023;23(1):809. DOI: 10.1186/s12913-023-09814-9 37507758 PMC10385916

[48] Harrison R, Walton M, Chitkara U, Manias E, Chauhan A, Latanik M, et al. Beyond translation: Engaging with culturally and linguistically diverse consumers. Health Expect. 2020;23(1):159–168. DOI: 10.1111/hex.12984 31625264 PMC6978859

[49] Wolfe I, Forman J, Cecil E, Newham J, Hu N, Satherley R, et al. Effect of the Children and Young People’s Health Partnership model of paediatric integrated care on health service use and child health outcomes: a pragmatic two-arm cluster randomised controlled trial. The Lancet Child & Adolescent Health. 2023;7(12):830–843. DOI: 10.1016/S2352-4642(23)00216-X 37866369

