# Supplementary material for: The Barriers and Enablers to Implementing Child and Family Health Hubs for Migrant Families
Source: Int J Integr Care. 2026 Jun 8;26(1):16. doi: 10.5334/ijic.9847 (PMC13262644; doi:10.5334/ijic.9847)
Supplement: Additional quotes. — Site A data to Site C data. [file ijic-26-2-9847-s1.pdf]

Additional quotes

Site A data

BUILDING BLOCK	PARTICIPANT QUOTE (PARTICIPANT ROLE)
**BUY IN**	We're evolving in the community to, by policy design or by mere effective changes in the community, to a service that's much more focused on vulnerable families. So I think it fits in the vulnerable family space… these families really, really need our help. (Health provider)
	Look at the latest AECD data, and you know, where some of our kids are in the [Site A] area, it's, it's terrifying. And the only way to invest in in making that better is to make help seeking easier. And if you've got a mum with three kids, and saying to her, "Oh, you've got to go to Waitara or whatever or Artarmon for whatever", they're just like, "what? - I'm not doing that I've got kids traffic school, I can't afford it, whatever". I think the responsibility is to develop those services inreach and outreach. (NGO manager)
	But I could imagine that with the childcare center, Child and Family Health, and us in this kind of governance group whatever you want to call it. Because at the end of the day, we're all wanting the same, we're wanting better outcomes. And all of us have a different role. So, no duplication. And if there'd be then that commitment, sure. Three Saturdays a year, we're going to have an open day, or, but that Child and Family Health can come. (NGO manager)
	Some amazing services within that geographical location. And some of those services work very close together, closely together. But it's also very fragmented as well. And they do still work in silos as well. So it's an underutilised concept, in my view (Health manager)
	One of the things that does and will make it easier, as I've just done it, is that the network that I certainly had, and the willingness of people to come on board as partners. I had to put this proposal together, and we got seven different agencies within a week, who said that they want to come on board in some way. And three of them said they wanted to co-locate. And I think, if we find the right place to co locate, it'll be more than that. (Health manager)
	So now have a look at the whole building… And trying to work with [some of the NGOs] is like pulling teeth and crawling over crushed glass to say, can we come to a staff meeting and can we remind you of what we do and what we don't do? I just don't know how you would shift the culture in this whole building (NGO manager)
**PARTNERSHIP**	Even though we've got this amazing service, the [NGO] on the top floor, got [NGO] on the next floor, we've also got an amazing supportive playgroup. A childcare center on the ground floor. We've also got housing in there as well. Child and Family Health is a little bit behind a closed door. And very clinician dependent on how and the relationship that they have with those other services within the same building. (Health manager)
	I think the Hub is like cohesiveness in how we interact with each other, so I feel like [NGO], we know the people upstairs, they know us… So we always recommend the [NGO] and people are happy as well to go there, because they trust us as well. (Health provider)
	I think that's the benefit, that flow of trust between agencies for the clients to be able to access, I also think that the fact that people

	know better what other agencies do means that they're more likely to refer and then most likely to remember to refer. (Health manager)
	Every six months, we do a Strengthening Partnerships for Community Good morning tea, where we get all of our co located partners, all of the partners in this building other services that sit around the area… But getting the other partners in this building to come is like pulling teeth… So we've just sort of pushed through and done our own and trying to drag the others along. (NGO manager)
	The other aspect of the Hub is collaboration and we used to have a wonderful community services officer who understood the interrelationship of services and understood the importance of having some coordination. So we did have a tenants' committee that was chaired by the community services, social planner, and that was amazing because his whole focus was collaborative practice. His whole focus was collaborative projects… And so we've had the most amazing collaborative practices. But now the council have said, well, we don't see much point in running that. And of course, now the whole thing is falling apart. And we might as well, once again, just be all separate people in a building, having nothing to do with each other. (Health provider)
	So I don't know the answer to your question, because they're all separate agencies. And I don't know who has, and I use the word power, because it has to be someone in a position who can access decision makers. And this is always the problem in you can have people come to a meeting, but unless they're in a position to access decision makers or make decisions or fund something themselves, all it is really is a - not all - It's an interagency collaboration, that's wonderful. But if you're trying to move something, then it has to be someone who can make a decision and can put funding behind it or resources (Health provider)
**HUB COORDINATION AND NAVIGATION**	I mean, just somebody to organize all the meetings, somebody to document the care pathways, and the agreed pathways, somebody to facilitate those conversations… Yes, definitely having a coordinator would definitely make it hang together a lot better than what typically happens in the interagency approach where everyone is stealing a bit of time. (Health manager)
	And, you know, if you were looking at anything, it's funding for coordination of interagency working groups that is needed, because I can see what will happen… everybody's motivated, but it's like well, have you got time to do it in your job? ….that coordination of that interagency networking, and working together gives what's needed. (Health provider)
	You need a coordinator. And part of that role of that coordinator would be working with those organizations to identify what the emerging need is, ensure that everyone's contracts are up to date etc. (NGO manager)
	I think they need a lead - somebody to call a coordinator of some description, somebody who would be available. So say, if there were regular case conferences, or multidisciplinary team meetings, you know, somebody who would be available to actually go to those (Health provider)
	I think there's a lot of work that has to happen with staff. Partly because community health staff in particular have got so used to probably extending their skill boundaries, more particularly things like early childhood, where, if there's not a social worker, will they start doing social work? …And the work that they do is partly because there just hasn't been people to refer so often they don't, but once you start working in colocations where the person's next door, what's to stop you. So I do think getting out of that "we have to do everything" it's takes a bit of culture change that you really have to work on. (Health manager)
	But it's it definitely is an exploratory pathway that even if there is leadership support, even if there are protocols to guide people, it's stuff that clinicians need a lot of support on, because then it's been so ingrained into us to keep people's stuff confidential and

	private. (Health manager)
	I think the majority of child and family health nurses want to work differently. But how do we make that transition? Because at the same time, many child and family health nurses see change as being quite threatening, and that letting go is part of this transition with the new release of... well, with all the resources that we're getting through Brighter Beginnings, is that going to be part of the the impetus to help that transition? Is it going to be when we get our new Safe Start policy when that comes out? That was meant to be out last year, but it's still not out. Will that be the it - you know what I mean? I just think there needs to be a reason for them to change (Health provider)
	I think, for instance, because if we're going to be a hub, will be classified as one, if we have like a reception staff and that way that can be someone coordinating, or the first point of contact is a reception that's universal to all the services (Health provider)
	[It's about a] single points of entry, or I like to think about it as a single process rather than a physical place. It may be one phone number, but it really doesn't matter who's answering that call (Health manager)
**RELEVANCE FOR MIGRANT AND REFUGEE FAMILIES**	So I think that partnership for general practice is super, super important. And I really think that for a lot of families, especially given that this project's about culturally linguistically diverse families, particularly, I mean, for all of us, there's a trusting relationship with a GP, if you've got a long standing relationship, we know from our GP pilot that some culturally linguistically diverse families will travel suburbs and suburbs and suburbs away to see a general practitioner who speaks their language. (Health manager)
	And [NGO] which is upstairs. They're amazing as well, because they have parenting groups. They have supported playgroups as well. And they get booked up so easily that people have to wait a whole term to go. And they also have a really lovely DV program they support with housing as well. And also [NGO]. So they do Circle of Security. So we're doing it in our group room at the moment. And that's a free course, which is so lovely. (Health provider)
	At [Site A] we have nurses who speak Mandarin and Cantonese, and Korean so we have fully qualified registered nurses with Child and Family Health Training who speak Korean Mandarin and Cantonese at that centre (Health provider)
	But can we make [the space] more appropriate, more approachable and warmer, and maybe some flags out the front - a bit of life and energy, have a concierge at the front desk to help you navigate your way through the building? …And you know how we can make it more alive and inviting, because it doesn't look like a community service. It looks like a Corporate Council Office. (NGO manger)
**ONGOING INTEGRATION**	At the moment, we've got a group email address for the collocated partners, so we can communicate to them one way. We have contracts with them - all of that sort of stuff, but we should be doing a little bit more reviewing of them. We should have someone here that actually does look at a client and goes, wow, this is working so well, this clients touched four services, as part of your model or this hub. (NGO manager)
	So we used to have tenants meeting every month, which was a really good meeting where there was information exchange, who's doing what, making sure we weren't duplicating that leveraging, you know, all of that sort of stuff was really good. But council pulled it. And we complained and sort of said, you know? And they basically said, Well, if you want one, you do it yourself. (NGO manager)
	So I think what is helpful as well, [NGO] gives us all their programs that they do as well. So we print out those and we hand them out. Same with the Relationships Australia, they give us their handouts, what they're going to do, and we hand them out to families that we feel will like them as well. (Health provider)

	A mother might come in, and she might say, “Oh, he’s really hard to settle all the time”. You know, a three month old – he’s pretty good, ticking all the boxes for development, and you're thinking what's going on. And then you get to the screening mothers for domestic violence, mental perspectives and all of that. And then you know, the mother decides she's going to talk to you and hasn't disclosed before she's got domestic violence. So we will actually, the family gets flagged, we're always very open with our families that we have to make a report explain benefits, explain the benefits of a DV card, we often walk them upstairs to [NGO] to actually meet the receptionist so that she can refer them to a caseworker. Still it should be easier than that. Because if I had said to a mother: “Look, as soon as you leave here, I've got another appointment straight after you. I want you to go upstairs. They are really nice and friendly. She'll have second thoughts just walking out the door. (Health provider)
	The other thing that's going to be important for general practice is to have a streamlined referral system to our services as well. And that's something that [Site A LHD] is getting very close to doing, having that central intake point as well. So GPs don't have to navigate that system, we can we can do that internally. (Health provider)
	We've been co located in that building for 9 or 10 years. And I don't see the type of integration at a client level, we would hope to achieve. I think there's a lot of good intent, but I don't think our service is particularly integrated at the client level. And I'm just going to reflect on a conversation as well around the previous building or tenants meeting that used to happen, again, that shared information about what different services are offering and what was on. I think that's an important part of the ingredient for a successful Hub. But on its own it doesn't lead to integrated care at a client level. (Health manager)

Site B data

BUILDING BLOCK	PARTICIPANT QUOTE (PARTICIPANT ROLE)
BUY IN	But I guess in terms of all of the services on site we’re, apart from the preschool who are preschool aged, we would really be the only child focused ones. But in saying that, we've got Parents Next on site here who are about supporting parents. And obviously, the housing support services as well. So I don't think things were overly clear to all of those programs as to why that we're being asked to be part of that more of a hub style for it as well. (NGO provider)
	[NGO]is very closely knit with the community nursing, family nursing health services that share one building very easily. (Project officer/Hub Coordinator)
	But no one's standing up saying we're doing it. They're just sort of saying “That doesn't affect me, I can't contribute”. And they're very quick to say “No, it's… there's nothing I can do”. (Project officer)
	It's [the NGOs] building, and they do their thing. And I don't think they really want to listen to what we have to say - in a nice way. They just feel that they're not required for the child and family health service. They think the ages that they see, the kids, have nothing to do with child and family health. (Health provider)
	[Initially] we had a program manager of [Site B NGO] pathway who was quite good with the whole [NGO] services, but her expectation of her role is to support me and to get me familiar with [NGO] parts not the health parts and she has no idea what happened to the health services like Child & Family Health Nurse, paediatrician, although she's an organiser, but she's quite busy.

	(Project officer/Hub coordinator)
	So I think for [NGO] the reality is that service providers they are very passionate about their role they want to collaborate and to provide their own clients more services. For example, a short waiting list to see a free paediatrician in the hub or some free assessment for the children if they have any developmental concerns, they are very, very happy. But on the Health side for child and family health services, I have some very deep conversation with them they feel that it seems: “Yeah it's good [NGO] has a lot of services but I prefer to refer my clients to [external hospital-based child and family service]”, because of the age range - a wide age range. [External hospital-based child and family service] can take a child from zero months but [NGO] only three years old sometimes. And also, for domestic violence they need to contact with a social worker rather than people at [NGO]. Or for playgroups, they provide the [local area council’s] playgroup information to the clients because there's no playgroups at [NGO]. So the child and family health service still do what they usually do in fact, but the good thing is that they know [NGO] if they are really a service that can meet the client's needs they definitely will do it. But till now - we have already got all the intervention group has delivered their babies - till now there is no referral happening inside [NGO] That's the reality. (Project officer)
	So the second part is what was totally different from my expectations about to the hub. So in fact at [Site B] the Hub it's kind of at the beginning, honestly speaking, I think there might be something wrong because it was not a hub when I first time arrived there, because during COVID it closed for a long time and a lot of service has been postponed or a lot of staff turned over and child family health centres close their clinic before the vaccination. And as the paediatrician team was short staffed. (Project officer/Hub coordinator)
	Not all of our partners, were actually appropriate to be part of the project, because one of them only delivers to primary school aged and the upper primary age children. So they weren't even sort of considered as part of it. Our family mental helath support service program is zero to eighteen. So that kind of fits. Our [psychological therapies for children] program is three to 12. So not hitting that target… But I guess in terms of all of the services on site we’re, apart from the preschool who are preschool aged, we would really be the only child focused one. But in saying that, we've got [parent support service] on site here who are about supporting parents. And obviously, the housing support services as well. So I don't think things were overly clear to all of those programs as to why that we're being asked to be part of that more of a hub style for it as well. (NGO manager)
**PARTNERSHIP**	Definitely, communication with all the service providers out there. We need to let them know that we want to restart this way that was our initial aim. A lot of members were saying that they don't get referrals, so they don't want to come [to meetings] because it's just a waste of time for them. That's when we opened up saying: “Okay, if you have cases, you bring those cases, and we need to work as a group to work out those cases”. “It's not only the patients that are coming in via the trial. Anyone can be bought into the … which is, I think, helpful in the way that we'll get more patients on board. The services can pitch in and sort of give their views as well. I think that's the main point I wanted to sort of say - we need to interact, and work as a team. (Project officer/Hub Coordinator)
	I guess my role feels quite segregated because as you just said, you do kind of feel like (especially because it's only once a month), you do kind of feel like you just turn up, you're busy and you let yourself into your room. You see your clients; you pack up and you leave. So there is that kind of disconnect where I wouldn't say that I feel like I'm part of the [Site B] Hub team, if that's the thing. I kind of feel like a contractor candidly. It's always nice to have more that feeling of being part of a team, but it's just it's yeah,

	in part, I think that barrier there is only being there once a month at the moment. (Health provider)
	If someone were to ask me, I guess I would say that it's [NGOs] building. And from my understanding, there's sort of multiple different health professionals that work out of there and provide a service from there. And I would say there's also the family daycare there as well. But aside from that, I don't actually know a lot more about it. And about the running of it (Health provider)
	I think the hubs are really good idea. I think integration is a really good idea. But I think that we are we have a very flawed model because we don't have the medical input. And it is a big, big, big flaw a lot of these hubs have worked on. I mean, a truly integrated service would have primary health care as the basis as the building block. (Health provider)
	I think just certain relationships. [The allied health provider] works out of here, she will come and eat lunch with us. And with my team, when she's on site here, which is absolutely amazing. As I said, I've moved into this role partway through the project. So it was really great to be able to develop a greater relationship with [CFH provider], whilst she was here. But I would say some of those bigger players within Health weren't at the meeting that [project officer] had organised. And by that, I mean more, the pediatricians. And I know, they are very time poor. We see that from the wait lists that they've got in terms of, you know, the children that we would like to refer, but those relationships just didn't seem to be forged. (NGO manager)
**HUB COORDINATION AND NAVIGATION**	I don't necessarily think that people were ready to have that shared collaboration and understanding. And I think that was really evident, even with the meetings that were set up in terms of attendance. So people coming to those meetings and some of the other [NGO] services weren't sure why they were there. But then, not everybody who was invited, and was supposed to be part of the hub was attending. So I think didn't thrive, partly for that reason, because it was hit and miss and sporadic. We had looked at it going online, one of the online meetings after [project officer] left… it ended up being Health jargon and talk and discussion. And it wasn't about the hub and what it was supposed to be. (NGO manager)
	I think [project officer] really had tried to hold that role together. And as I said, it really seemed to fall apart once she had moved on from the project. Because she really had coordinated everybody, trying to coordinate those meetings, and she'd done an amazing job at it. That would certainly be something that would be needed ongoing to make a successful hub is to have a key, key driver, coordinator to make that happen. And then it's that one person who is then having that shared understanding, getting everybody on the same page, etc, to make those connections that haven't happened as well, (NGO Manager)
	I don't know if I'm going to work any different, I'm late in the game in my profession, here so we work independently as clinicians too. So it was an experience working [within the Hub]… it was good in the sense that the mothers utilised coming. And I'm glad that will show in the research that mothers stayed with the project. So I hope there is a benefit that comes out, but we need to look at the service provision, like from [NGO] I mean, because that's the difference in what they have running to what, you know, the age group. (Health Provider)
**RELEVANCE FOR MIGRANT AND REFUGEE FAMILIES**	I think that the emerging populations move into Site B because of the cheapness of rent, [which] then leads itself to the inequities of trying to access interpreters at those times that they're being seen. I know it has been a problem, but also just trying to get the message across, about the importance of child and family to some of these emerging populations is difficult. I think the emerging populations have come from cultural backgrounds where free is not good. And so, having to pay for things that they can't afford is seen to be better than accessing something that's free... And, the availability of a GP that might speak their own language that might be open five days a week, open on a Saturday morning, open late Thursday nights, particularly with the culturally and

	linguistically diverse populations. A lot of these women don't drive. And so they're relying on their husbands to get them there. Or a significant other family member. That's often after hours. (Health Provider)
	My concern is [Site B] will struggle with engagement. Look, and some things have turned around simply because there's a strong service and a leader and a person who really connects up well. And again, we don't have that… I think there needs to be a good link with primary care because I think the engagement with child and family health nurses is not strong for [the local migrant and refugee] population. (Health provider)
	In [Site B], the migrant population has very basic English literacy… because they are newly migrants sometimes when you ask for information, they think they might be charged, or they may think: “You are you're from the government” (Project officer/Hub coordinator)
	It wasn't really suitable [for migrant and refugee families with babies under 12 months] … But a lot of times, the mums utilise the GP and some are already utilising a paediatrician from within the hospital, are already referred. So, I think sometimes that didn't quite fit with the mothers, or a lot of times they didn't have a lot of issues. It's more like breastfeeding. You know, initially, or bottle feeds. We looked at that from the Child and Family point of view. And other services weren't available for that group, like counselling, they didn't really have for under 12 months. So, what was good was the nutritionist, dietician and that I thought was ideal, because we did do some referrals. Not a lot, but we did utilise it. (Health provider)
**ONGOING INTEGRATION**	No, I don't have much to do with them at all. It's been a little bit hard for me to attend all their meetings. I did go to one recently, and I haven't really had a lot of referrals from them. I think one of the good things about my project that people do find appealing, is that I do have childcare. So the childcare most of the time is in the same room as where I am with their mum. And the parents love that I have childcare, because they can actually sit and they can talk to me. I know that the only real conversation that I had with a [health provider] was that she could have childcare. But I haven't really had many referrals [from the health providers] (NGO provider)
	In terms of [health], we don't typically have that kind of warm referral. And really, we don't get a lot of referrals, even across our mental health programs. And with the partners for that, zero to two, even zero to three space within our demographic, I don't think a lot of those families are out there and being seen. So we seem to find the younger children from… we might be seeing a child and it's a younger sibling. So the warm referrals do happen within sort of existing services, probably not so much with [newer health providers] (NGO manager)
	I think that was the purpose initially, of the meetings that [project officer] had set up - to be some of those collaborative case conferences and discuss: “I've got this child or this family that I'm working with, I think this is what they need. How can the hub best support that” It didn’t happen…. I think that comes back to people's understanding of what those meetings were and commitment to be part of that - those meetings and that process. (NGO manager)
	Part of it sort of felt like there was this expectation of: “What can [NGO] bring and what can [NGO] add?” But without asking, “What can you bring and add to this?” So it was like, [project team] says, “Yeah, we're happening. Everyone come and meet together”.

	And then things really didn't change along the way… I think that clear picture of what the hub was, or was supposed to be, it wasn't really defined. Yeah, we all want to get on the same page. Some people got onto that page. But as I said before, if people aren't attending those meetings, then what's the purpose? (NGO manager)
	I think some very open conversations would be kind of a really good first step. And putting out some really clear expectations. Because as I said to you, we have those conversations with our partners, they do their warm referrals across each other as well. So it would be trying to integrate that with Health. And we will all need to see ourselves not in our little individual little silos, and understand what we can do for each other. I think that's the other breakdown in it that there is no, clear: “this is what we can do to connect and support each other”. (NGO manger)
	Well, I just feel like the once a month meetings, it became much more difficult after [senior health manager’s] passing. (Health provider)

Site C data

BUILDING BLOCK	PARTICIPANT QUOTE (PARTICIPANT ROLE)
**BUY IN**	There's a high need, higher need in this area for support for children, preschoolers, especially that might have delays in different parts of their development that they need extra support for before they start school. (Health provider)
	I guess we have a lot of families, where the children - some developmental delays, not hitting their milestones, and a lot of the triggers in the beginning before they start school. So it was important that we have people who were able to help us help our families access the right people, say, to go through a paediatric assessment, where they might not be able to get anywhere else to get their four year old checklist, you know, their blue book, and making sure that they're meeting all the milestones, if they're not, then having someone who can refer them on to things that we may not have access to. (NGO provider)
	Unless we had [the Hub], there would be a significant proportion of the population that would be missed and missing that early intervention. And then therefore turning up at school, not ready for school and not, you know, not being able to live their best life. (Health provider)
	And I think you get as much from it as what you put into it. So if you're willing to make the connections with people and, you know, you're really committed to giving your best to the kids and the families, then you get so much out of it. (NGO provider)
	I don't think we ever set out to start a hub, what we set out to do was, I suppose move our services to work with an NGO that these families were actually connecting to so they trusted that service, they trusted that venue, they were willing to go to that venue. So I suppose we moved our services there we went very much with we're just going to collocate. And the hub has been something that's progressed, or that notion of a hub has been something that I suppose developed after that pilot after that pilot project, because it was really successful. (Hub coordinator)
	[Site C] is high needs space, there's a lot of CALD clients living here. Over the years, there's been an influx of like Bangladesh, Nepalese clients, and overseas visitors and people that don't have Medicare, and that can't access a lot of services unless they pay for it. So, and there's a high need, higher need in this area for support for children, preschoolers, especially that might have delays

	in different parts of their development that they need extra support for before they start school. (Health provider)
**PARTNERSHIP**	We now have started to introduce a… it's not an MOU, it's like a service agreement…it basically really just outlines what, if you come on as a partner, this is what it actually means. (Hub coordinator)
	I guess it's always a choice of whether you want to work in that model or not. And I think you want to reach people that wanted you to work in that model. You don't want to be dragging people to work in that model…you have to give 100% … and if you're not prepared to do that, it's not going to work. (Health provider)
	Do not make changes without asking everyone who's a participant in this. Because at the end of the day everyone is putting effort and their own time into this. At the end of the day, this is all free work. (NGO provider)
	So it basically really just does outlines what if you come on as a partner, this is what it actually means. This is what we expect. You know, we expect you to turn up to a working group meeting, we expect you to participate in at least one activity, we expect you to do what we call warm referrals. (Hub coordinator)
**HUB COORDINATION AND NAVIGATION**	You need someone who is in tune with all the subtleties that often are completely missed. I think without that everyone is busy, and everyone gets back into their own organisations. I think without someone who brings you together all the time it's hard to keep the momentum going. (NGO manager)
	We haven't had completely dedicated funding for that and that has been one of the losses we've had in the Hub because we've kind of stacked it onto people's jobs and that's always been a challenge. (Health manager)
	It's nice having that one person who knows and is able to control what's going on and explain. She's only working two days or three days whatever it is, I think you need to have that person that's sort of knows what's going on throughout the service, throughout all the areas so that they can send people off. We know there is this one person we can say: "Hey, this has happened. What do we do?" (NGO provider)
	My role is a bit of a mash up of several different people's roles. So because we leased the building on the school site, I'm now the office manager, basically where everything from order toilet paper to you know, get the air conditioner replaced, to deal with the school around work health and safety. But that's more about the nature of the building that we have here. Although if you were in a commercial premises, you would still have to have a building manager as well. But whether that would be the role of the coordinator or they would give that to someone else might be different. (Hub coordinator)
	It's basically simply finding a pathway to get them to the hub or to other services, sitting down with them, and having a clear conversation about what it is that they are looking for and what are the needs of your child, you as a family, and you in the community and how can we meet those needs. What are the support services available for you and who can help you immediately and where will you be on a waiting list, what are the different forms that you need to fill out, who might you need to speak to, will language be a barrier, where I can help you at all times, I can attend things with you as well, I can make the calls for you. Just to simplify the process for them so it's not as frightening as we know it is for them. (NGO provider)
**RELEVANCE FOR MIGRANT AND REFUGEE FAMILIES**	Making sure that women and families have a choice of what they want to access and when and that's part of a mapping exercise. And the navigator once again supports them to say what services are provided at the different locations and their benefits and sorting the referral. (Health manager)
	We always provided opportunities for interpreters and translated information, and I think I had a good understanding of working with

	that community for quite a number of years now in that role. I was aware of their cultural religious practices, and that certainly helped. That understanding and training.” (Health provider)
	[Interviewer: Are you attending any other child and family health services within the Hub?] No, no, I'm just attending the GP when [the baby] needs. Otherwise, she's healthy. (Mother)
	what would you say about your experience with the services you're receiving at [the Hub]. Oh, yeah they're nice. Everything is nice with me and they are like attending to me very well… they have classes and everything in my language as well. (Mother)
	I can only see benefits [of the Hub]. I just think it's a one stop shop. It's just so holistic - one of our CFH Nurses works there and, I haven't spoken to her, but even looking at the time that they're allocated, the families are given extra time with the nurse. So that means you can explore everything in more detail. There's the availability of interpreters too, which always when you're working with an interpreter takes time. A lot of these families too are from non English speaking backgrounds. And so like that child that I saw, it would have been great if they'd been seen sooner, but they didn't have that same standard of health care as us. The [Site C] Hub - having that whole multidisciplinary team, and the availability of like speech, occupational therapy, dental vision, like the Child and Family Health Nursing, they've got the pediatrician, too that goes there. It's just amazing. So I can only see benefits for that, especially for those vulnerable communities. (Health provider)
**ONGOING INTEGRATION**	Part of that commitment is it's about warm referrals. It's about giving feedback. It's about having a key person within each of those partner organisations that you can reach out to at any time and ask a question. I think that's really important. (Hub coordinator)
	I knew that there was collaboration and referrals from partners in the Hub, but I didn't realize how much administration work was involved, and feedback, which is a very positive thing. (Health provider)
	Yes, the difference being that at [Site C Hub], in mainstream service, we would see our clients and then refer off to allied health. And that would probably be the end of it in terms of feedback from the client, etc. However, in the Hub, we have partners that actually refer to me with children that they're concerned about. And then I will feed back information to the referrer as well. And then we sort of integrate with our partners, so that we feed back to one another, and we also get referrals from the preschool, childcare or the public school, which is next door to us that have concerns about children that are starting kindergarten the next year. So yeah, that's kind of the main differences. (Health provider)
	So referrals, I'm not sure. Like, it doesn't even necessarily have to be from the Hub, its from people that are part of the Hub, or they were already involved. So I'm not sure it's a direct process. It's just the connection like we said. But it's just the connections. It's for me, it's more about the relationship that you develop with people in the hub, and your contact people. And yeah, that rapport and being able to ask questions and discuss clients in cases with that personal touch that you wouldn't have had otherwise. (NGO provider)
